# Lipocalin-2 may produce damaging effect after cerebral ischemia by inducing astrocytes classical activation

**DOI:** 10.1186/s12974-019-1556-7

**Published:** 2019-08-19

**Authors:** Nan Zhao, Xiaomeng Xu, Yongjun Jiang, Jie Gao, Fang Wang, Xiaohui Xu, Zhuoyu Wen, Yi Xie, Juanji Li, Rongrong Li, Qiushi Lv, Qian Liu, Qiliang Dai, Xinfeng Liu, Gelin Xu

**Affiliations:** 1 Department of Neurology, Jinling Hospital, Medical School of Nanjing University, 305 Zhongshan East Road, Nanjing, 210002 Jiangsu China; 2 Department of Neurology and Institute of Neurology, Ruijin Hospital, Shanghai Jiao Tong University School of Medicine, 197 Ruijin Er Roud, Shanghai, 20025 China; 3 Department of Neurology, The Second Affiliated Hospital of Guangzhou Medical University, 250 Changgang East Road, Guangzhou, 510260 China

**Keywords:** Astrocytes, Functional polarization, LCN2, iNOS, Classical activation, Ischemic stroke

## Abstract

**Background:**

Functions of astrocytes in the rehabilitation after ischemic stroke, especially their impacts on inflammatory processes, remain controversial. This study uncovered two phenotypes of astrocytes, of which one was helpful, and the other harmful to anoxic neurons after brain ischemia.

**Methods:**

We tested the levels of inflammatory factors including TNF-a, IL-6, IL-10, iNOS, IL-1beta, and CXCL10 in primary astrocytes at 0 h, 6 h, 12 h, 24 h, and 48 h after OGD, grouped the hypoxia astrocytes into iNOS-positive (iNOS(+)) and iNOS-negative (iNOS(−)) by magnetic bead sorting, and then co-cultured the two groups of cells with OGD-treated neurons for 24 h. We further verified the polarization of astrocytes in vivo by detecting the co-localization of iNOS, GFAP, and Iba-1 on MCAO brain sections. Lentivirus overexpressing LCN2 and LCN2 knockout mice (#024630. JAX, USA) were used to explore the role of LCN2 in the functional polarization of astrocytes. 7.0-T MRI scanning and the modified Neurological Severity Score (mNSS) were used to evaluate the neurological outcomes of the mice.

**Results:**

After oxygen-glucose deprivation (OGD), iNOS mRNA expression increased to the peak at 6 h in primary astrocytes, but keep baseline expression in LCN2-knockout astrocytes. In mice with transient middle cerebral artery occlusion (tMCAO), LCN2 was proved necessary for astrocyte classical activation. In LCN2 knockout mice with MCAO, no classically activated astrocytes were detected, and smaller infarct volumes and better neurological functions were observed.

**Conclusions:**

The results indicated a novel pattern of astrocyte activation after ischemic stroke and lipocalin-2 (LCN2) plays a key role in polarizing and activating astrocytes.

## Significance statement

This study uncovered two phenotypes of astrocytes, of which one was helpful, and the other harmful to anoxic neurons after brain ischemia. Lipocalin-2 (LCN2) plays a key role in polarizing and activating astrocytes. After oxygen-glucose deprivation (OGD), iNOS mRNA expression increased to the peak at 6 h in primary astrocytes, but keep baseline expression in LCN2-knockout astrocytes. This suggests that LCN2 may be an effective molecule for post-stroke intervention. Inhibition of LCN2 can prevent astrocytes from activated in a harmful way and reduce the neurological damage in the acute phase of stroke and improve the prognosis after stroke. The results indicated a novel pattern of astrocyte activation and a potential therapeutic target for ischemic stroke.

## Highlights

1. Astrocytes may function as inflammation mediators after brain ischemia.
2. Polarization may endow astrocytes varied functions in the survival of anoxic neurons.
3. LCN2 may harm the functional recovery of neurons from ischemic injury by promoting classical activation of astrocytes.

## Introduction

Astrocytes in the brain can be activated via two typical pathways after injuries, of which one is usually regarded as helpful, and the other as harmful [1]. Functional polarization is a new concept mainly applied to macrophages [2] and has been intensely investigated in microglia in recent years [3]. Under inflammatory conditions, classically activated macrophages, known as M1 cells, can produce pro-inflammatory factors like nitric oxide (NO) and tumor necrosis factor-alpha (TNF-α). The alternatively activated macrophages, known as M2 cells, can produce anti-inflammatory factors like interleukin-10 (IL-10) and arginase-1 (ARG1) [4–6]. Astrocyte, stimulated by lipopolysaccharide (LPS) or interleukin-4 (IL-4), can also function as inflammation mediator via a process called phenotypic polarization [7]. The impacts of astrocyte phenotypic polarization on central nervous system (CNS) infectious diseases have been investigated extensively [1], but seldom on ischemic stroke.

Lipocalin 2 (LCN2), a member of the lipocalin family, is an acute-phase protein with multiple functions [8]. LCN2 is synthesized and released in CNS after inflammation, infection, or injury [9, 10]. LCN2 can induce a variety of chemokines to participate in inflammatory responses [10–12] and may determine the phenotypes of macrophages. In mice experiment, LCN2 increased gradually and peaked at 23 h after cerebral infarction mainly in astrocytes and endothelial cells [13]. Some studies demonstrated that LCN2 could exert neuroprotective effect after CNS inflammatory diseases and brain injury [9, 12]. Other studies, however, demonstrated that LCN2 could exert neurotoxic effect after ischemic and hemorrhagic stroke [14–16]. LCN2 may upregulate glial fibrillary acidic protein (GFAP) via Rho–ROCK (Rho kinase)–GFAP pathway and then change cell morphology after inflammatory stimulation [17]. But the functions of LCN2 in ischemia-induced astrocytes activation are still controversial. To our knowledge, this is the first study to investigate the impacts of astrocyte polarization and the roles of LCN2 on astrocyte polarization after brain ischemia.

## Methods and materials

### Animal

Male C57BL/6 mice of 8–10 weeks old were used for in vivo experiments. Wild-type (WT) mice were provided by the Model Animal Research Institute of Nanjing University (Nanjing, Jiangsu, China), and LCN2 knockout (KO) mice (B6.129P2-Lcn2tm1Aade/AkiJ, Jax, 024630) were provided by the Jackson Laboratory and housed in a specific pathogen-free facility in Model Animal Research Center of Nanjing University. All animal experiments followed the Guide for the National Institutes of Health Guide for the Care and Use of Laboratory Animals (NIH Publications No. 8023, revised 2011) and were approved by the Animal Care Committee (Institute of Science and Technology, Jiangsu Province, China). Animals were maintained on a 12-h light/dark cycle with free access to food and water. Invasive procedures were performed under isoflurane anesthesia (RWD, Shenzhen, China) to minimize the animal suffering.

### Animal model of brain ischemia

Transient middle cerebral artery occlusion (tMCAO) was performed following previously reported procedures [18]. After right external carotid artery ligation, a small incision was made on the right common carotid artery and a silicone-coated 6–0 nylon monofilament advanced through the common carotid artery and the internal carotid artery to reach the opening of the right middle cerebral artery and to cause middle cerebral artery occlusion. Cerebral blood flow was monitored with Laser Doppler flowmeter (LDF; Perimed PF5000, Stockholm, Sweden) throughout the procedure. A reduction in blood flow of over 70% was considered as surgical success. After 90 min (or other ischemic time indicated), the monofilament was withdrawn to allow reperfusion.

### Neuron culture

Primary cortical neurons were obtained from fetal mice of embryonic day 18–20, as previously reported [19]. After careful removal of meninges, cortical tissues were digested in Hank’s balanced salt solution (HBSS, Gibco) containing 0.125% trypsin (Gibco) at 37 °C for 15 min. Dulbecco’s modified Eagle medium (DMEM, Gibco) containing 10% FBS (Gibco) was then added to terminate the digestion. The mixture was homogenized by pipetting 60 times and was subsequently filtered with a 100-μm cell strainer (Biologix) and centrifuged at 200*g* for 5 min. Then, the cells were collected and resuspended by DMEM supplemented with 10% FBS (Gibco) and penicillin-streptomycin (Gibco). The suspension was seeded into 6-well plates (106 cells/ml), and 2 h later, the medium was changed with Neurobasal medium (Gibco) containing 1% L-Glutamate and 2% B27 supplement. At the third day, 1/3 of the medium was changed and the neurons were used at the sixth or seventh day.

### Astrocyte culture

Neonatal mice within 24 h after birth of either sex were used to obtain mixed glial cultures as previously documented [20]. Briefly, cerebral cortices of the pups were isolated in HBSS on ice and meninges were removed. The tissue was then digested in 0.125% trypsin at 37 °C for 15 min before DMEM with 10% FBS was added. The mixture was homogenized by pipetting 100 times and then passed through cell strainer and centrifuged at 200*g* for 5 min. The cells were re-suspended by DMEM containing 10% FBS and penicillin-streptomycin and seeded in cell flask (Costar) coated with Poly-L-Lysine (Sigma) overnight. The medium was changed 24 h later to remove non-adherent cells. After that, half of the medium was changed every 3 days. When the mixed cell culture reached confluence, astrocytes were isolated by shaking the flasks at 37 °C at 300 rpm for 4–6 h. The adherent cells were washed by PBS twice and dissociated by 0.25% trypsin for 2 min at 37 °C. Subsequently, the cells were centrifuged at 200*g* for 5 min and then seeded in cell culture dishes (Costar). Half of the medium was renewed every 3 days.

### Oxygen-glucose deprivation

Oxygen-glucose deprivation (OGD) model was established in accordance with the previous protocol [19, 20]. Briefly, after replacing the culture medium with glucose-free DMEM (Gibco), the cells were placed into a sealed chamber, which was then flushed with 95% N_2_ and 5% CO_2_. After 2 h for neurons or 6 h for astrocytes, the glucose-free medium was replaced with the initial cultural medium, and the cells were returned to the normoxic incubator for 0-, 6-, 12-, 24-, or 48-h reperfusion.

### RNA tests

RNA was extracted using TRI Reagent (Sigma) from brain tissues or cultured cells following the manufacturer’s instructions. In brief, the tissue samples or cells were homogenized with TRI Reagent, and chloroform was added at a 1:5 ratio (TRI Reagent: chloroform = 1:5). After thorough mixing, the suspension was centrifuged at 4 °C, 12000 rpm for 15 min. Then, the upper layer was mixed with an equal volume of isopropanol and stored at − 20 °C for 30 min. Finally, the samples were centrifuged and dissolved in RNase-free water after washing by 75% alcohol. RNA quality was determined by A260/280 with BioTek Epoch (SN253825). For each sample, 200 ng total RNA was used for reverse transcription using RevertAid First Strand cDNA Synthesis Kit (Thermo Scientific). Subsequent polymerase chain reaction (PCR) was performed in a Mx3000P Real-Time PCR System (Agilent Technologies) with UltraSYBR Mixture (CWBio) and following primers in Table 1.

**Table 1:** DNA sequences of the primers used for RT-PCR

Mouse cDNA		Primer sequences
Tnf-alpha	Forward	5′-CCTCCAGAAAAGACACCA-3′
	Reverse	5′-ACAAGCAGGAATGAGAAGAG-3′
Il-6	Forward	5′-GGTATAGACAGGTCTGTTGGGAG-3′
	Reverse	5′-CTTCTTGGGACTGATGCTGGTGA-3′
Il-1ra	Forward	5′-ATAGTGTGTTCTTGGGCATC-3′
	Reverse	5′-CGCTTGTCTTCTTCTTTGTT-3′
Inos	Forward	5′-GTCCTACACCACACCAAACT-3′
	Reverse	5′-ATCTCTGCCTATCCGTCTC-3′
Il-1beta	Forward	5′-AAGTTGACGGACCCCAAAAGAT-3′
	Reverse	5′-TGTTGATGTGCTGCTGCGA-3′
Cxcl10	Forward	5′-ATCCTGCTGGGTCTGAGT-3′
	Reverse	5′-CATCTCTGCTCATCATTCTTT-3′
Il-10	Forward	5′-AGTGAACTGCGCTGTCAATG-3′
	Reverse	5′-TTCAGGGTCAAGGCAAACTT-3′
Lcn2	Forward	5′-CTCAAGGACGACAACATCA-3′
	Reverse	5′-CACACTCACCACCCATTC-3′
Gapdh	Forward	5′-AAGAAGGTGGTGAAGCAGG-3′
	Reverse	5′-GAAGGTGGAAGAGTGGGAGT-3′
Arg-1	Forward	5′-CGCCTTTCTCAAAAGGACAG-3′
	Reverse	5′-CCAGCTCTTCATTGGCTTTC-3′
Arg-1	Forward	5′-AATGGAAGAGTCAGTGTGGT-3′
	Reverse	5′-GTTGTCAGGGGAGTGTTG-3′

### Immunofluorescence staining

Immunofluorescence staining was performed with cultured cells and 14-μm-thick frozen brain sections, as previously reported [18, 19]. In brief, the cells or brain sections were fixed in 4% PFA (pH 7.0) for 10 min. After washing with phosphate-buffered saline (PBS), the slides were immersed in 0.3% Triton X-100 (Sigma) for 30 min and 0.5% skim milk for 1 h before they were incubated in primary antibody overnight at 4 °C. On the second day, the slides were washed with PBS containing 1% Tween and incubated with secondary antibody in dark for 2 h. Finally, the slides were stained with DAPI (Sigma) for 10 min, mounted with CC/MOUNT (Sigma), and imaged with FluoView FV10i confocal laser scanning Microscope (Olympus). The primary antibodies are as follows: mouse anti-GFAP (1:400, Cat#MAB360, Millipore), goat anti-GFAP (1:200, Cat#ab53554, Abcam), rabbit anti-iNOS (1:1000, Cat#ab17945, Abcam), rabbit anti-ARG1 (1:1000, Cat#ab124917, Abcam), rabbit anti-beta-actin (1:2000, Cat#4970, Cell Signaling Technology), mouse anti-beta-III-Tubulin (1:300, Cat#ab78078, Abcam), rabbit anti-24p3R (1:200, Cat#ab124506, Abcam), goat anti-LCN2 (1:200, Cat#AF1857, R&D). The secondary antibodies are as follows: anti-mouse Alex 488 conjugated secondary antibody (1:200, Cat#ab150117, Abcam), anti-rabbit Alex 488 conjugated secondary antibody (1:200, Cat#ab150073, Abcam), anti-goat Alex 594 conjugated secondary antibody (1:200, Cat#ab150132, Abcam), anti-rabbit Alex 647 conjugated secondary antibody (1:200, Cat#ab150063, Abcam), anti-rabbit secondary antibody (1:2000, Cat#14708, Cell Signaling Technology).

### Western blotting

Brain tissue samples and cells were lysed with RIPA lysis buffer (Cell Signaling) containing 1 mM phenylmethanesulfonyl fluoride on ice for 15 min and then centrifuged at 14,000 g for 15 min. Supernatant was collected and the concentrate was quantified by BCA protein assay kit (Thermo Scientific). After denaturation with loading buffer at 95 °C for 5 min, protein samples were separated on 7.5% and 10% SDS-PAGE gels at 85 V, 30 min followed by 105 V, 90 min, and transferred onto polyvinylidene difluoride membranes (Millipore) at 250 mA, 2 h. The membranes were blocked with 5% skim milk (Generay, Shanghai, China) at room temperature for 2 h, and incubated with primary antibodies in blocking buffer at 4 °C overnight. On the second day, the membranes were reacted with HRP-conjugated secondary antibody for 1 h at room temperature. Bands were detected using Enhanced Chemiluminescence kit (Merck Millipore, Merck KGaA, Darmstadt, Germany) in the dark, according to the manufacturer’s protocol.

### Neuroimaging

Magnetic resonance imaging (MRI) examination was carried out using a 7.0-T MRI scanner (BRUKER PharmaScan, Germany). Mice were anesthetized with isoflurane and their body temperature was maintained at 37.0 ± 0.5 °C with a heating pad. For each animal, 15 T2-weighted images (T2-WI) of 0.5 mm-thick (repetition time/echo time = 2500/36.0 ms, 256 × 256 pixel) and 15 slices of diffusion-weighted images (repetition time/echo time = 5000/22.0 ms, 256 × 256 pixel) matching T2-WI images were acquired.

### Fluoro-Jade C staining

Fluoro-Jade C staining (FJC) was performed with cultured cells according to the manufacturer’s manual (Millipore). The cells were immersed in basic alcohol solution (1% NaOH in 80% ethanol) for 5 min, and then transferred into 70% ethanol for 2 min. The slides were immersed in 0.06% potassium permanganate solution for 10 min before being incubated in 0.0001% working solution of FJC for 15 min. After washing and drying, slides were cleared in xylene for 1 min before cover-slipped with neutral balsam.

### TUNEL staining

Terminal deoxynucleotidyl transferase-mediated dUTP nick end labeling (TUNEL) staining was carried out using the In Situ Cell Death Detection Kit, AP (Roche, USA) according to the manufacturer’s protocol. After sequentially immersed in 4% PFA for 20 min and 15% glacial acetic acid for 2 min, the slides were incubated with the reaction solution at 37 °C for 2 h, then converter-AP for 30 min. Slides were washed and slightly dried before mounted with CC/MOUNT.

### Magnetic labeling

Primary astrocytes were counted and washed with an excess volume of 1X BD IMag™ buffer, and the supernatant was carefully aspirated. Anti-iNOS primary antibody was added, mixed thoroughly, and incubated at room temperature for 30 min. Then, the second antibody bind with magnetic particles was vortex thoroughly and added into the system. The magnetic labeling volume was adjusted to 1–8 × 10^7^ cells/ml, and the tube was immediately placed on the BD IMagnet™, incubated for 8–10 min. With the tube on the BD IMagnet™, the supernatant which contained the negative fraction was carefully aspirated off. Then, the tube was removed from the BD IMagnet™ and washed twice for 2–4 min. After the final wash, the positive fraction was resuspended in an appropriate buffer or media.

### Flow cytometry

Adherent cells were digested with 0.25% trypsin at 37 °C for 2 min, and the digestion was terminated with medium. The cells were collected by centrifugation at 1500 rpm for 5 min. The cells were washed twice with PBS (discarded the supernatant after centrifugation at 1500 rpm for 5 min) and resuspended. The cells were then treated with 1% Triton-100 and incubated at 37 °C for 10 min. Antibody was added and mixed according to the manufacturer’s instructions. After being incubated at 37 °C for 30 min in the dark, cells were washed twice and then resuspended in 400 μl of PBS. The cell phenotype was tested with flow cytometry in 30 min.

### Statistics

Infarct volumes were determined using ImageJ (1.49v, NIH, USA). Statistical analysis was performed with SPSS (24.0v, IBM, USA). Parameters were expressed as mean ± SE. Comparisons were carried out with Student’s *t* test or one-way ANOVA followed by Fisher’s least significant difference (LSD) post hoc test, as appropriate. Statistical significance was deemed as *P* < 0.05.

## Results

### Involvement of astrocytes in hypoxia-induced inflammation

**Fig. 1:**
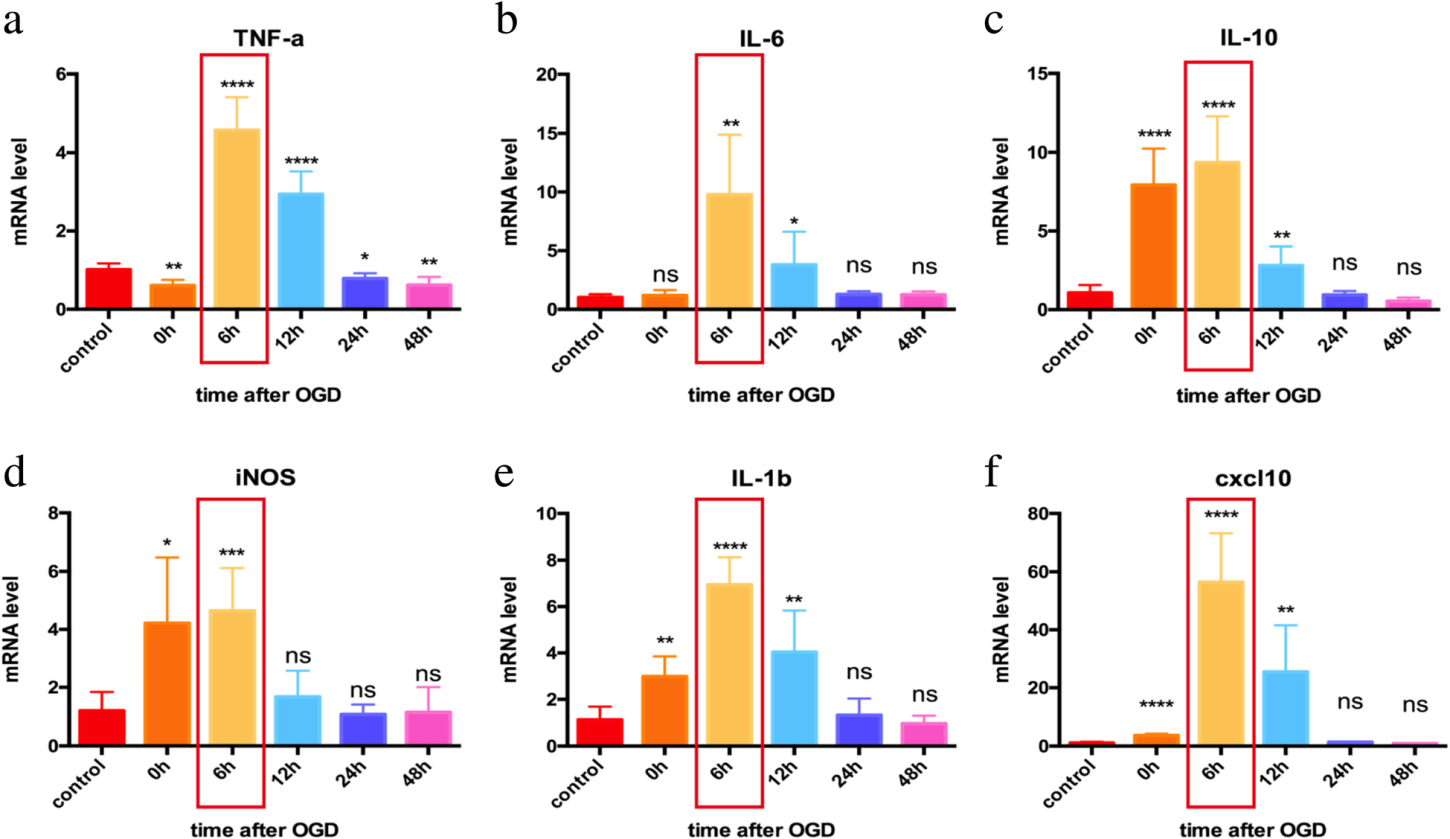
Expressions of several typical inflammatory factors in primary astrocytes after OGD. **a**–**f** The primary cultural astrocytes were harvested 0 h, 6 h, 12 h, 24 h, and 48 h after OGD for 6 h. RT-PCR was used to detect the mRNA levels of TNF-a, IL-6, IL-10, iNOS, IL-1b, and CXCL10. The mRNA of all these cytokines were elevated in 6 h after ischemia (*P* < 0.05) and decreased after 24 h

**Fig. 2:**
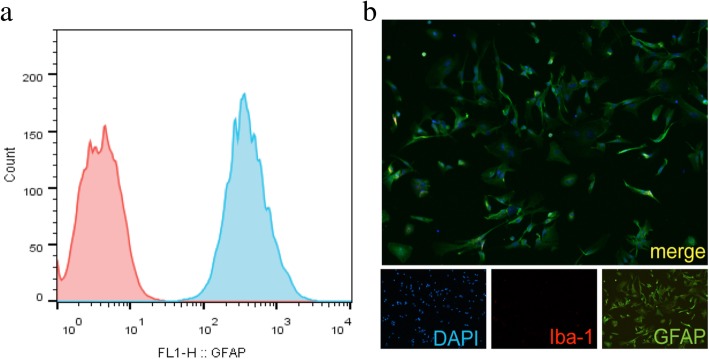
Purity of primary astrocytes certification. **a** The primary cultured astrocytes were labeled with fluorescent GFAP antibody. Flow cytometry was used to detect the purity of primary cultured astrocytes. There were more than 99% cells labeled with GFAP. **b** Primary cultured astrocytes in slides were labeled GFAP antibody binding fluorescence, DAPI fluorescent labeled nuclei. Slides were observed under the fluorescence microscope. More than 98% cells were GFAP labeled (scale bar 100 μm). Antibody: mouse anti-GFAP (1:400, Cat#MAB360, Millipore), anti-mouse Alex 488 conjugated secondary antibody (1:200, Cat#ab150117, Abcam)

In order to explore the roles of astrocytes in post-stroke inflammation, the levels of inflammatory factors were detected in primary astrocytes at 0 h, 6 h, 12 h, 24 h, and 48 h after 6-h OGD, including TNF-α, IL-6, IL-10, inducible nitric oxide synthase (iNOS), IL-1β, and CXCL10 (Fig. 1, Table 1). The mRNA levels of TNF-α, IL-6, IL-10, iNOS, IL-1β, and CXCL10 increased significantly at the sixth hour after OGD and returned to the baseline levels after 24 h, suggesting that astrocytes participated in the inflammatory responses to hypoxia by increasing the expression of inflammatory factors. Flow cytometry and immunofluorescence tests showed that the astrocyte purity was over 99.7% (Fig. 2), indicating that the increased inflammatory factors were expressed by astrocytes, rather than by possibly contaminating microglia.

### Polarization of astrocytes after OGD

**Fig. 3:**
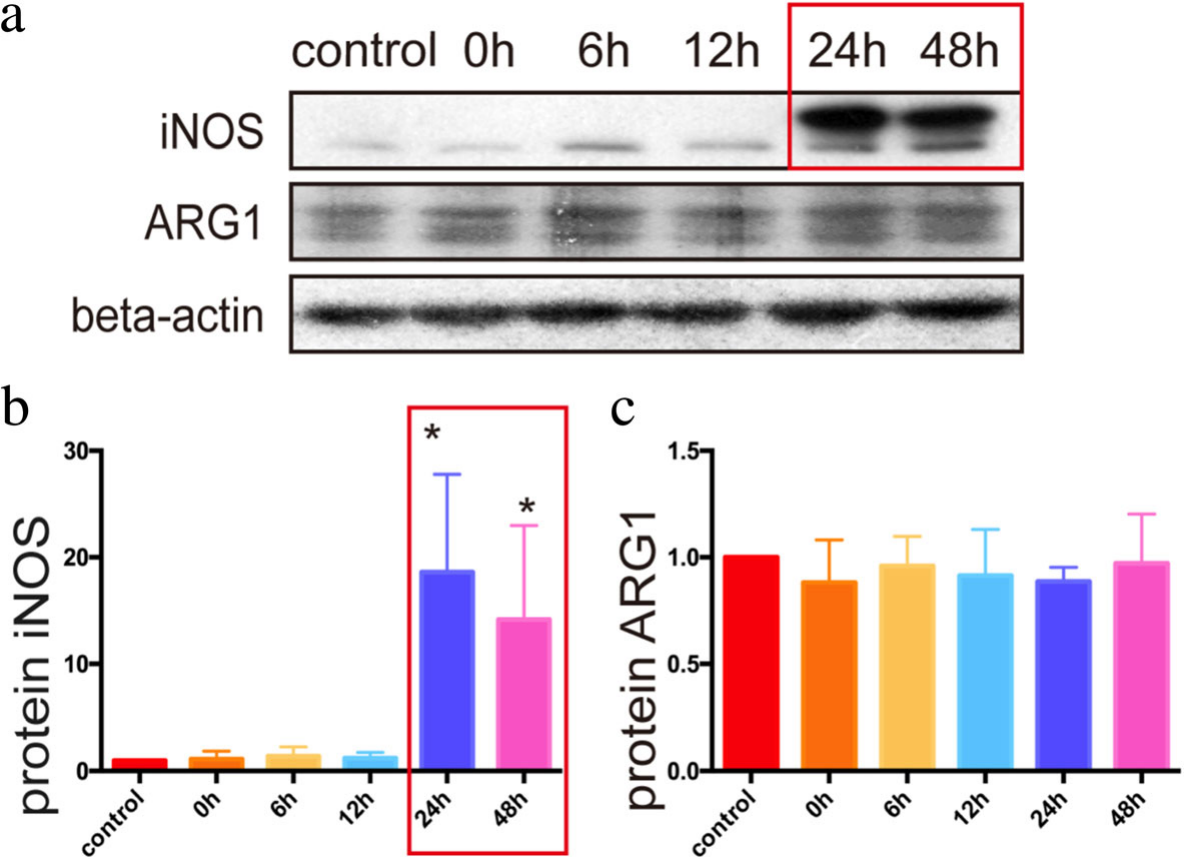
The iNOS and ARG1 expression and anoxic astrocyte activation. **a** The primary cultural astrocytes were harvested 0 h, 6 h, 12 h, 24 h, and 48 h after OGD for 6 h. Western blot was used to detect the expression of protein iNOS and ARG1. **b** The expression of iNOS was hardly detected in 0 h, 6 h, and 12 h after OGD, but had an over 15 times increasing after 24 h (*p* < 0.05). **c** The expression of ARG1 did not change significantly after OGD. **b**, **c** The statistical results of primary cultural astrocytes detected by Image J. Antibody: rabbit anti-iNOS (1:1000, Cat#ab17945, Abcam), rabbit anti-ARG1 (1:1000, Cat#ab124917, Abcam), rabbit anti-beta-actin (1:2000, Cat#4970, Cell Signaling Technology), anti-rabbit (1:2000, Cat#14708, Cell Signaling Technology)

The functional polarization of macrophages was well-acknowledged, with iNOS and ARG1 as markers for M1 and M2, respectively [21, 22]. Therefore, in our study, we also selected iNOS and ARG1 as markers and detected their expression levels in cultured astrocytes after OGD. The results showed that iNOS increased sharply at 24 h after OGD (1.000 ± 2.092e−006: 18.61 ± 5.299, *p* = 0.0293), while the level of ARG1 did not change significantly (Fig. 3).

**Fig. 4:**
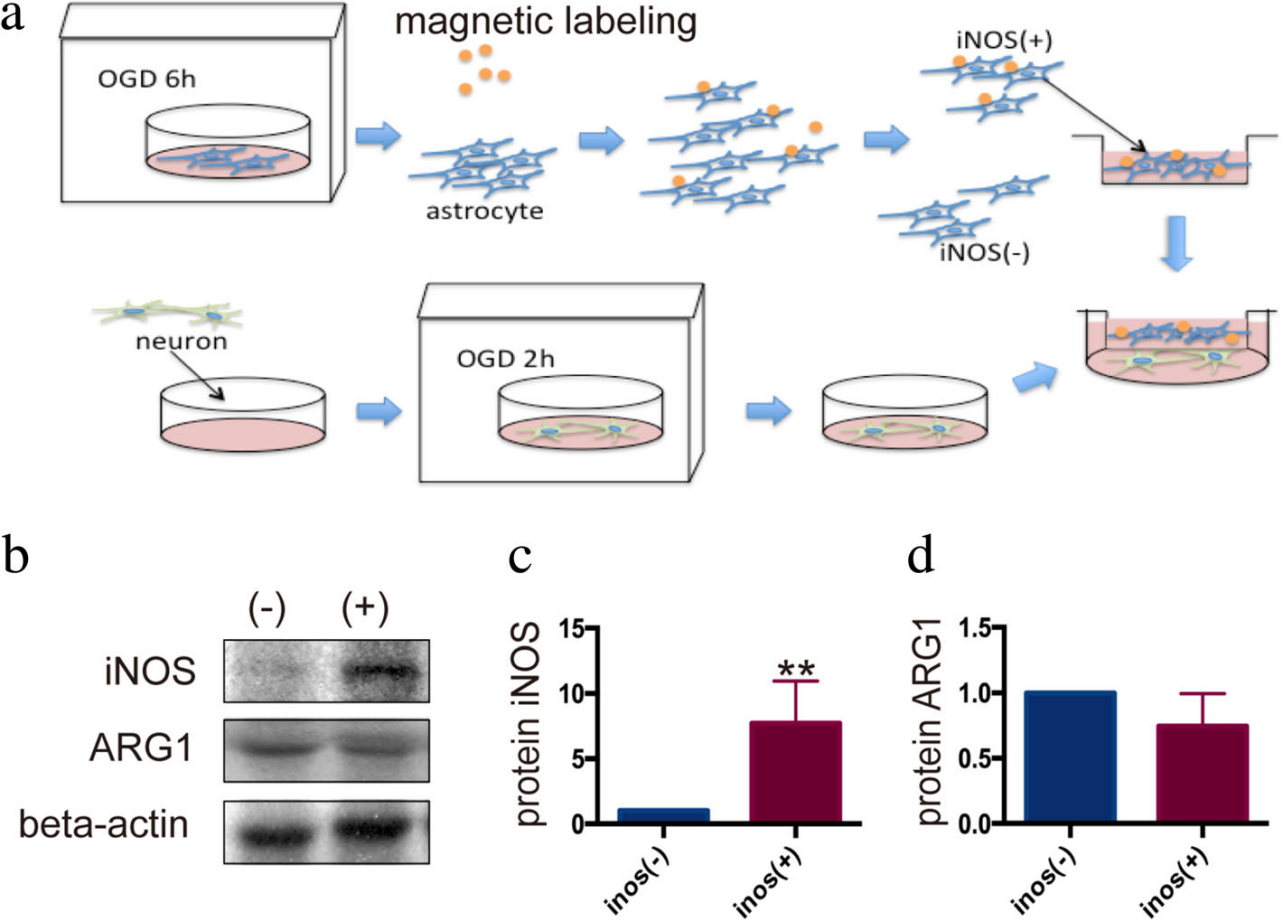
Anoxic activated astrocytes sorted according to iNOS expression. **a** The astrocytes were harvested 24 h after OGD and were grouped according to the expression of iNOS by magnetic particle labeling. **b** Western blot was used to detect the expression of protein iNOS and ARG1 in the two groups. The two groups had a significant difference in iNOS expression (*P* < 0.01) but had no difference in ARG1 expression (*P* = 0.52). **c**, **d** The statistical results of Image J. Antibody: rabbit anti-iNOS (1:1000, Cat#ab17945, Abcam), rabbit anti-ARG1 (1:1000, Cat#ab124917, Abcam), rabbit anti-beta-actin (1:2000, Cat#4970, Cell Signaling Technology), anti-rabbit (1:2000, Cat#14708, Cell Signaling Technology)

**Fig. 5:**
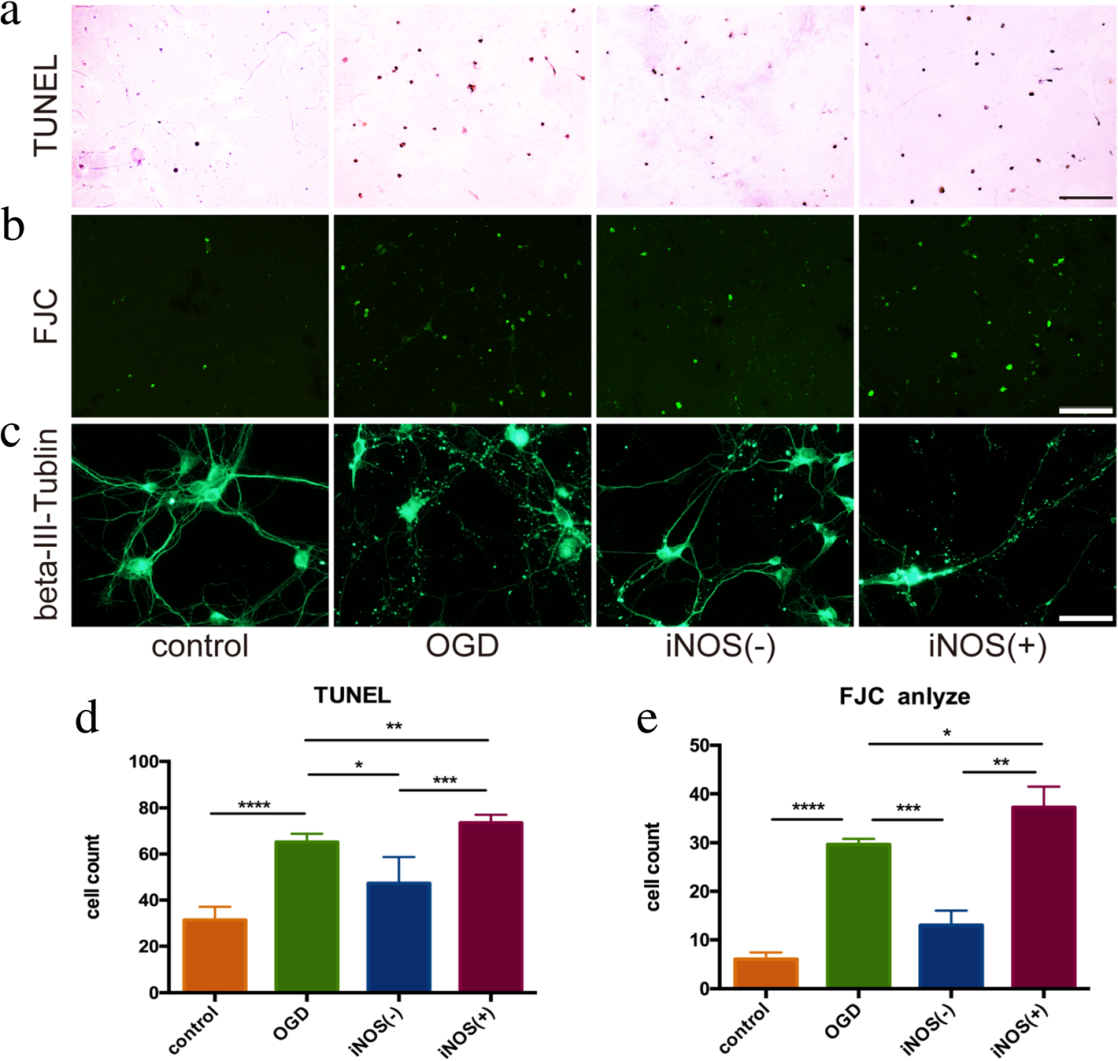
Effects of two types of astrocyte activation on anoxic neurons. The anoxic neurons were co-cultured with the two groups of astrocytes separated by magnetic particle label. TUNEL (**a**) and FJC (**b**) were used to detect the apoptosis and denaturation of neurons, with scale bar of 50 μm. **d** The statistical results of TUNEL showed that the anoxic neurons had more apoptosis than control group (*P* < 0.001); the iNOS(+) group had more apoptosis than the anoxic neurons group (*P* < 0.01), while the iNOS(−) group had less apoptosis than the anoxic neurons group (*P* < 0.05). **e** The denaturation of each group was parallel to apoptosis. **c** Immunofluorescence staining was used to observe the morphological structure of neurons. The structure of the synapse was damaged in the anoxic neuron group. Neurons in iNOS(+) group were relative integrity, while the neurons in iNOS(−) group were more seriously damaged, scale bar 10 μm. Antibody: mouse anti-beta-III-Tubulin (1:300, Cat#ab78078, Abcam), anti-mouse Alex 488 conjugated secondary antibody (1:200, Cat#ab150117, Abcam)

Therefore, in subsequent experiments, we chose iNOS as a marker and demonstrated that the astrocytes after hypoxia treatment can be grouped into iNOS-positive (iNOS(+)) and iNOS-negative (iNOS(−)) by magnetic bead sorting (Fig. 4a). The difference in iNOS expression between the two groups of astrocytes was further verified by WB (Fig. 4b–d). We then co-cultured the two groups of cells with OGD-treated neurons for 24 h. The neurons co-cultured with iNOS(+) astrocytes showed increased apoptosis and degeneration after OGD. Neuron apoptosis increased by 13.6% (*P* = 0.004), and neuron degeneration increased by 25.8% (*P* = 0.0372). The apoptosis and degeneration were significantly ameliorated among neurons co-cultured with the iNOS(−) astrocytes. Neuron apoptosis reduced by 25.4% (*P* = 0.014), and neuron degeneration reduced by 56.2% (*P* = 0.0009, Fig. 5).

**Fig. 6:**
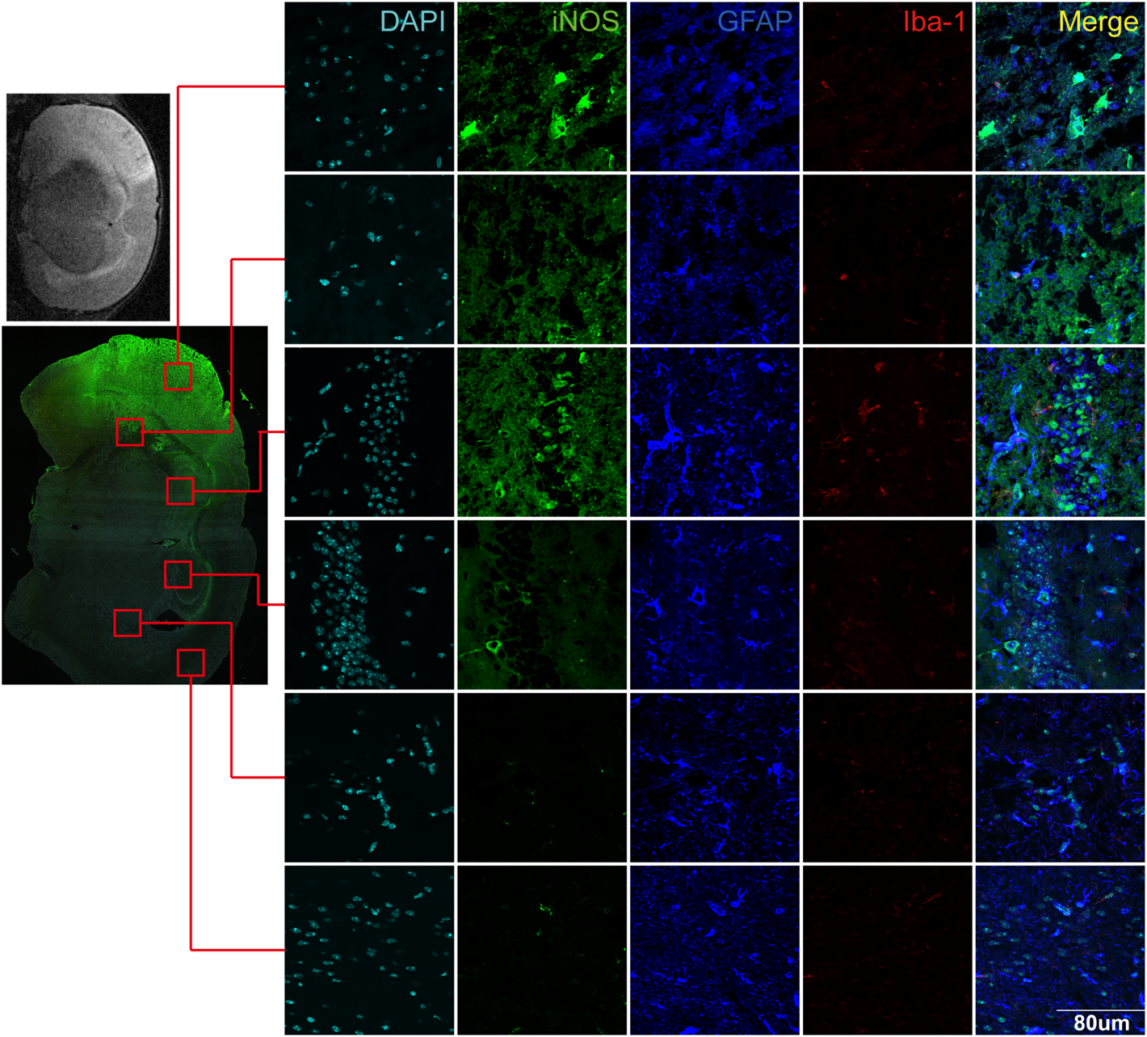
Co-localization of iNOS, Iba-1, and GFAP in MCAO mouse brain section. The mice were sacrificed 24 h after MCAO. Immunofluorescence stain was used to test the co-localization of iNOS, Iba-1, and GFAP in the MCAO mouse brain section. The iNOS (green) was mainly expressed in the infarct area and not expressed in the healthy area in the contralateral hemisphere. The iNOS protein was co-localized with GFAP (blue), but not with Iba-1 (red). Antibody: mouse anti-iNOS (1:200, Cat#ab49999, Abcam), anti-mouse Alex 488 conjugated secondary antibody (1:200, Cat#ab150117, Abcam), goat anti-GFAP (1:200, Cat#ab53554, Abcam), anti-goat Alex 647 conjugated secondary antibody (1:200, Cat#ab150135, Abcam), rabbit anti-Iba-1 (1:400, Cat#016-20001, Wako), anti-rabbit Alex 594 conjugated secondary antibody (1:200, Cat#ab150064, Abcam)

We further verified the polarization of astrocytes in vivo by detecting the co-localization of iNOS, GFAP, and Iba-1 on MCAO brain sections. The expressions of iNOS in the central region of infarct cortex, marginal region of infarct cortex, and hippocampus were significantly higher than those in the corresponding regions of the contralateral cerebral hemisphere. The increased expression of iNOS was mainly co-localized with GFAP, an astrocyte-specific marker. On the contrary, iNOS and Iba-1 were seldom observed in the same cell, and some GFAP-positive cells were not positively stained with iNOS (Fig. 6). This suggests that astrocytes also have polarization after ischemia and hypoxia in vivo.

### The roles of LCN2 in astrocyte polarization

**Fig. 7:**
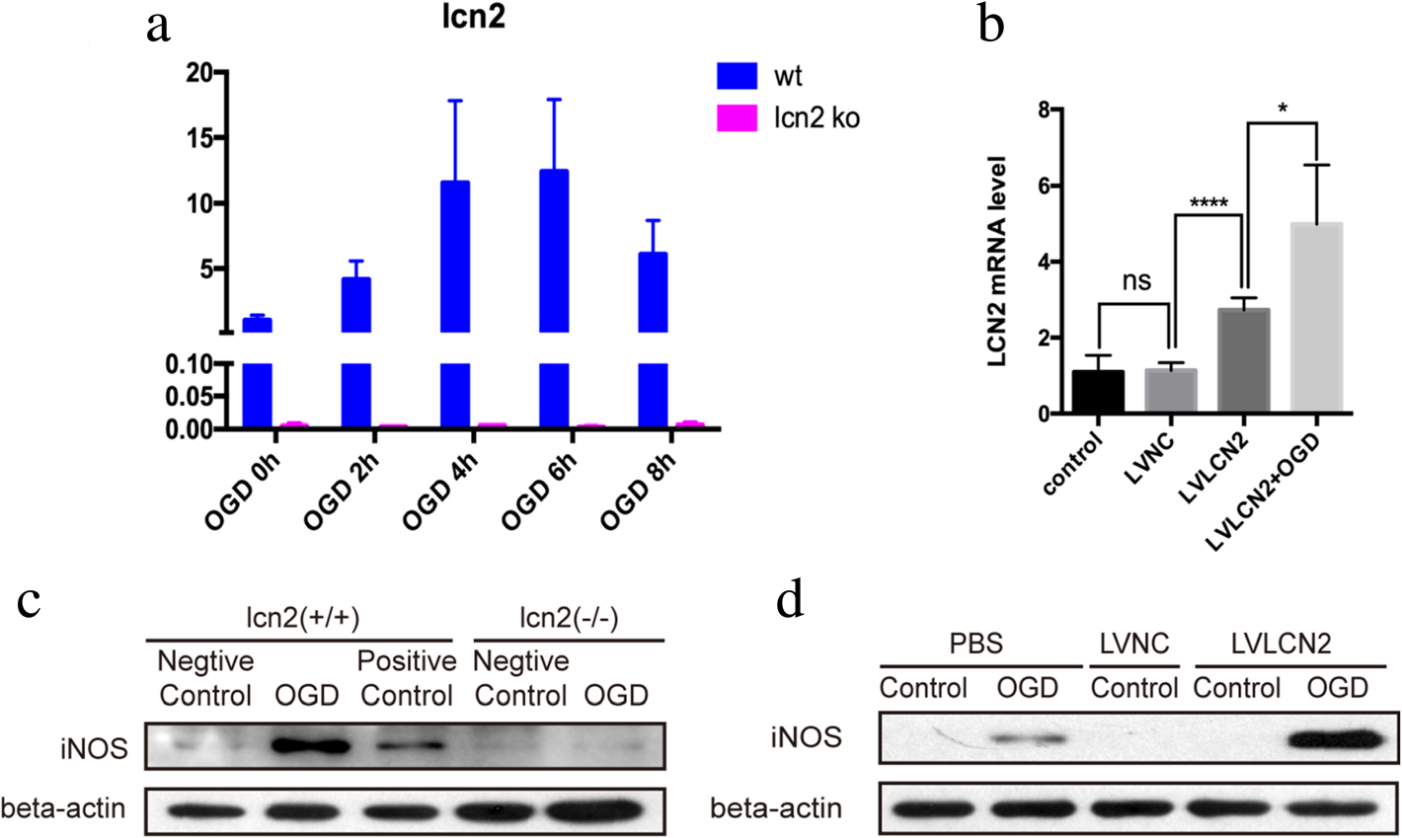
Expression of iNOS after LCN2 knockout and lentivirus transfection. **a** The WT and LCN2 knockout astrocytes were harvested 24 h after OGD for 0 h, 2 h, 4 h, 6 h, and 8 h. RT-PCR was used to detect the mRNA levels of LCN2. Compared with WT control, the LCN2 mRNA stayed no expression in LCN2 knockout astrocytes. The LCN2 mRNA level of WT astrocytes reaches up to over 10 times after 6 h OGD treatment. **b** RT-PCR was used to detect the mRNA levels of LCN2 after lentivirus overexpressed LCN2 transfection (LVLCN2) and its negative control (LVNC). The LVLCN2 treatment can increase the expression of LCN2 mRNA (*P* < 0.0001), and the LVNC had no effect on LCN2 expression (*P* > 0.05). **c** Astrocytes were harvested 24 h after 6 h OGD. Western blot was used to evaluate the protein iNOS level. The iNOS level increased sharply after OGD in WT astrocytes, but there was no iNOS expression in LCN2 knockout astrocytes with or without OGD treatment. **d** Astrocytes were transfected by LVLCN2 for 6 h and treated by OGD 24 h later. Astrocytes were harvested 24 h after 6 h OGD. The Western blot showed that anoxic induced iNOS expression in transfected astrocyte increased greatly. Antibody: rabbit anti-iNOS (1:1000, Cat#ab17945, Abcam), rabbit anti-beta-actin (1:2000, Cat#4970, Cell Signaling Technology), anti-rabbit (1:2000, Cat#14708, Cell Signaling Technology)

In order to explore the role of LCN2 in the functional polarization of astrocytes, lentivirus overexpressing LCN2 and LCN2 knockout mice (#024630. JAX, USA) were used as gain-of-function and loss-of-function approaches. Primary astrocytes from knockout mice did not express LCN2 at both physiological condition and different time points after anoxia stimulation, confirming the complete deletion of LCN2 (Fig. 7a). Although the expression of 24p3R, the receptor of LCN2 protein, was not significantly affected by LCN2 knockout, no LCN2 expression was observed on the brain slices of knockout mice after MCAO surgery (Fig. 10). The results of RT-PCR demonstrated that the transfection of lentivirus successfully increased the expression of LCN2 mRNA (Fig. 7b).

**Fig. 8:**
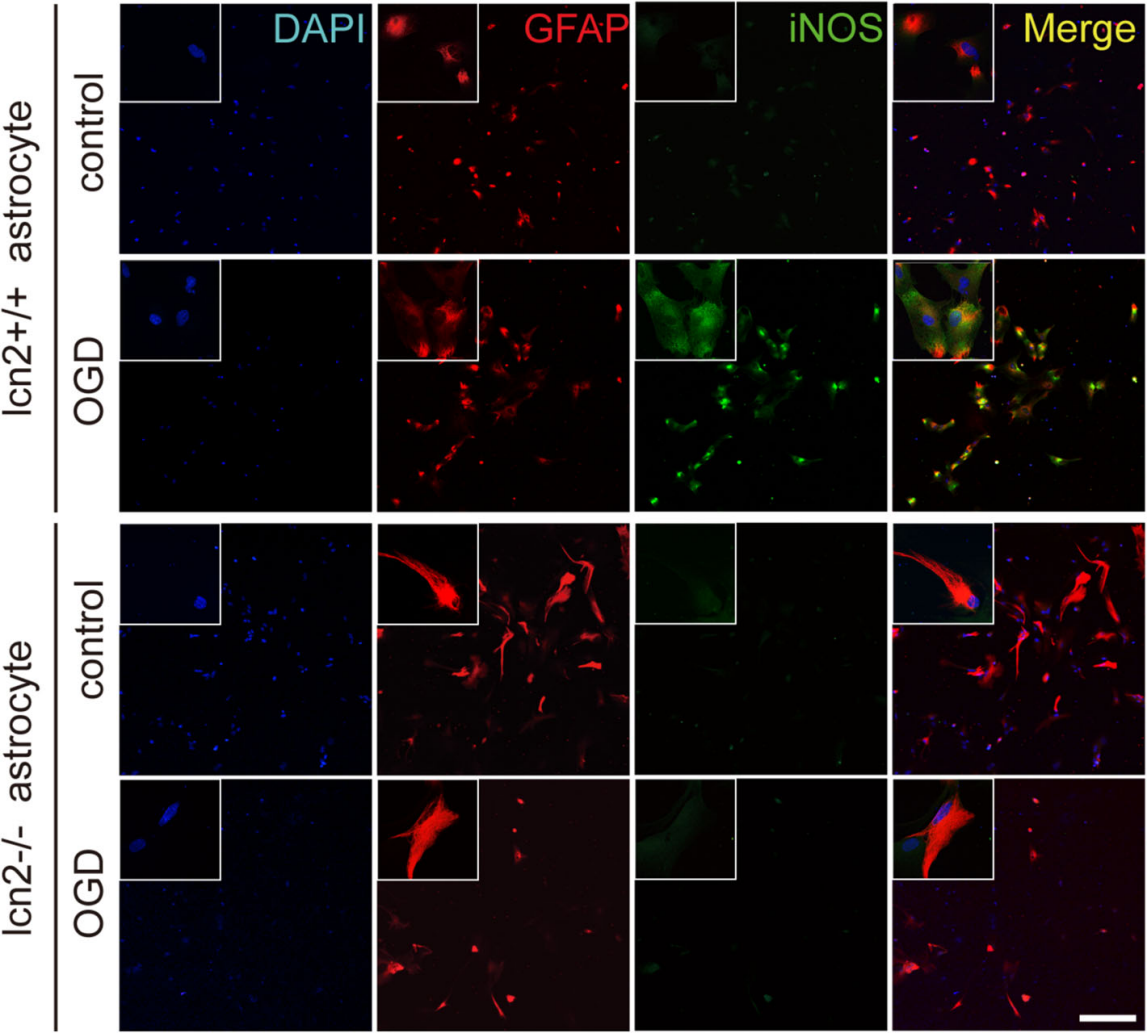
Inhibition of OGD induced iNOS expression in LCN2 knockout astrocytes. The iNOS and GFAP were co-localized in primary cultured wild-type astrocytes after oxygen-glucose deprivation. iNOS did not express in LCN2 knockout astrocytes after oxygen-glucose deprivation, scale bar 200 μm. Antibody: rabbit anti-iNOS (1:200, Cat#ab17945, Abcam), anti-rabbit Alex 488 conjugated secondary antibody (1:200, Cat#ab150073, Abcam), goat anti-GFAP (1:200, Cat#ab53554, Abcam), anti-goat Alex 594 conjugated secondary antibody (1:200, Cat#ab150132, Abcam)

**Fig. 9:**
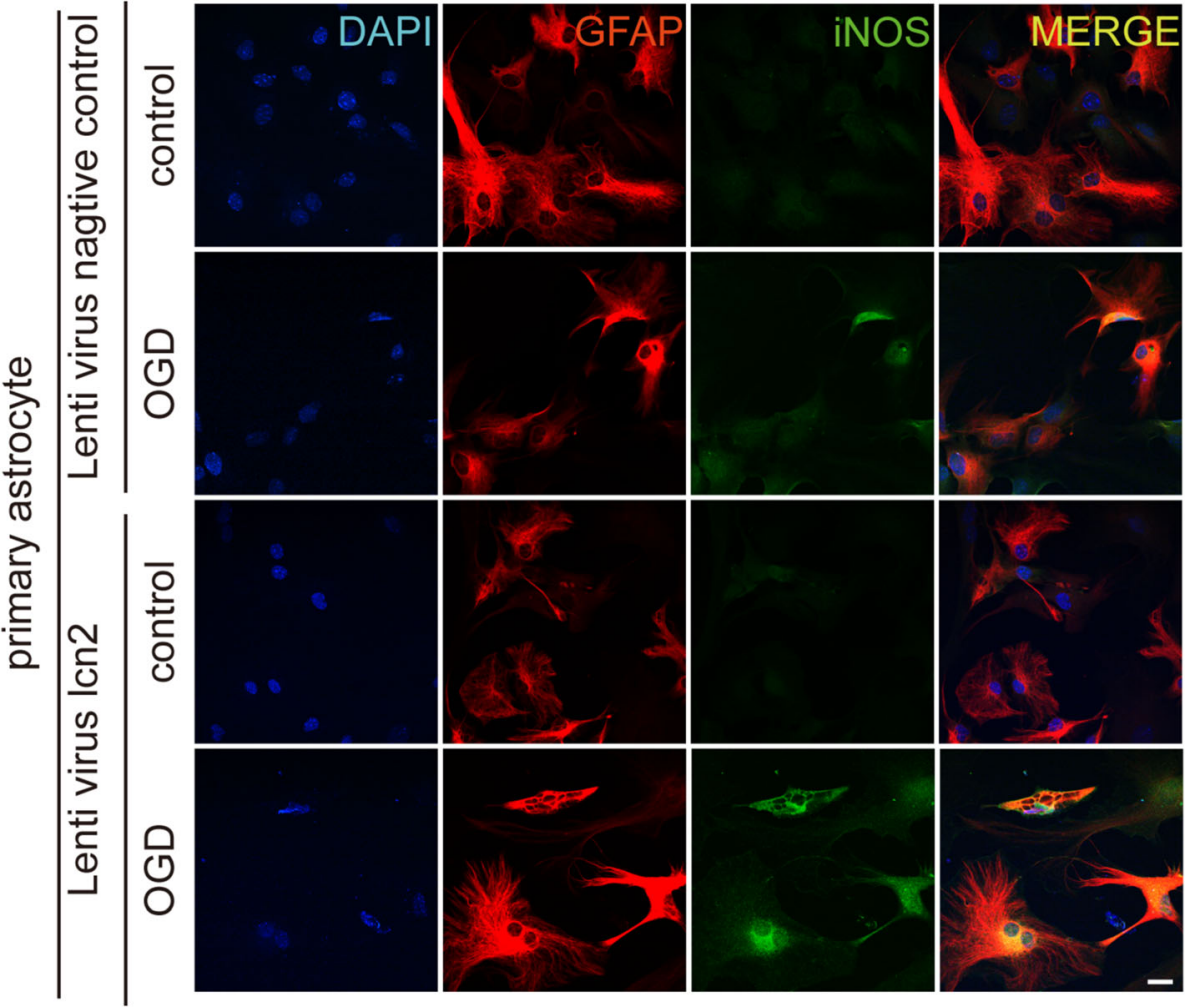
Increase of OGD induced iNOS expression in LCN2-lentivirus transfected astrocytes. Astrocytes were transfected by LVLCN2 for 6 h and treated by OGD 24 h later. Astrocytes were fixed at 24 h after 6 h OGD. The immunofluorescence showed that iNOS expression was increased in LCN2 overexpressed astrocytes after oxygen-glucose deprivation, scale bar 20 μm. Antibody: rabbit anti-iNOS (1:200, Cat#ab17945, Abcam), anti-rabbit Alex 488 conjugated secondary antibody (1:200, Cat#ab150073, Abcam), goat anti-GFAP (1:200, Cat#ab53554, Abcam), anti-goat Alex 594 conjugated secondary antibody (1:200, Cat#ab150132, Abcam)

**Fig. 10:**
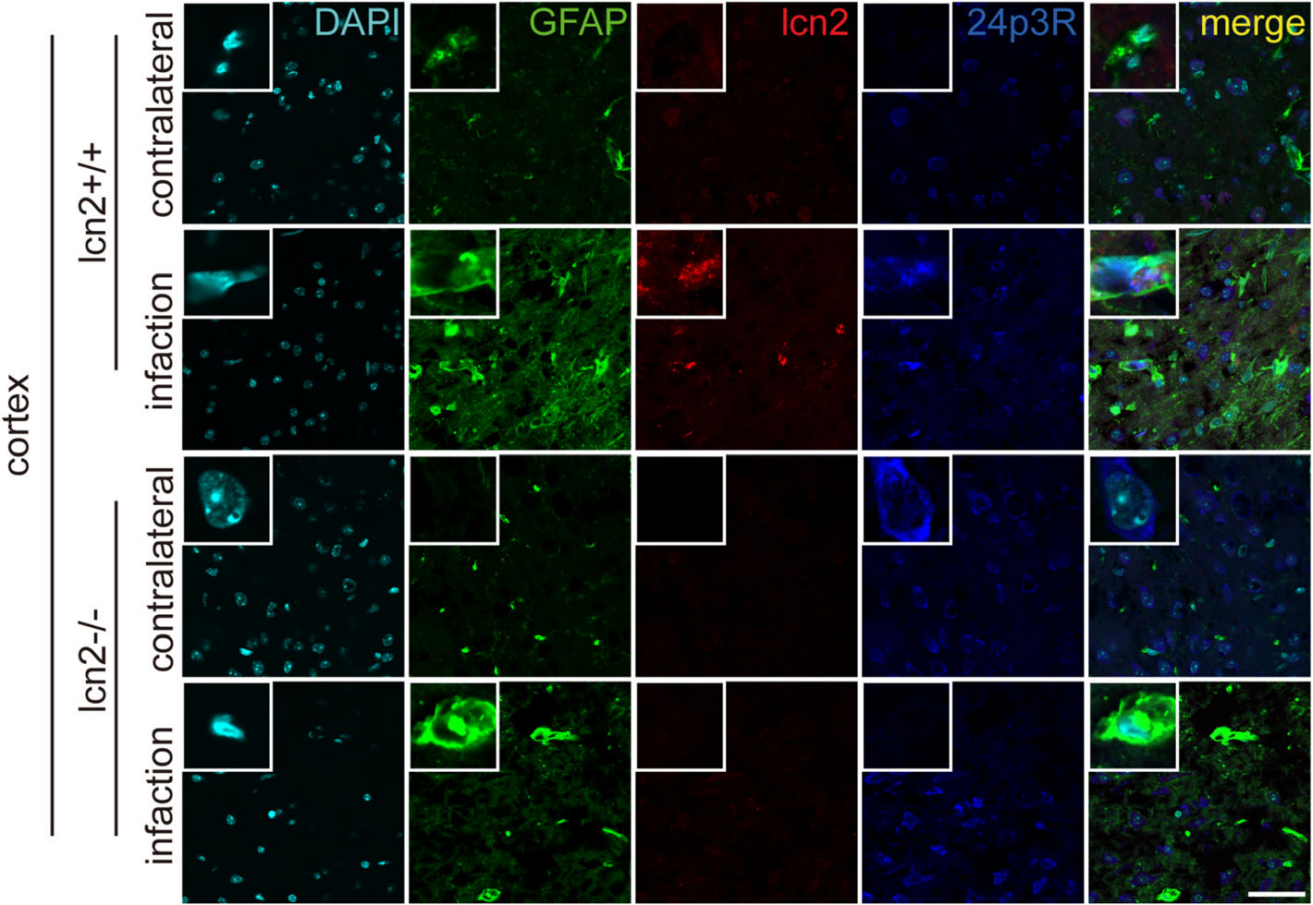
Expression of LCN2 in brain section of MCAO mice. The protein LCN2, its receptor 24p3R, and GFAP were co-localized by immunofluorescence on brain slices of MCAO mice. LCN2 was overexpressed in the infarcted area. Co-localization of 24P3R and LCN2 was not observed in the contralateral area. The 24p3R was widely expressed throughout the brain and was not affected by MCAO. LCN2 protein was no longer detected after LCN2 knockout, but the expression of 24P3R was not changed after LCN2 knockout, scale bar 40 μm. Antibody: mouse anti-GFAP (1:400, Cat#MAB360, Millipore), anti-mouse Alex 488 conjugated secondary antibody (1:200, Cat#ab150117, Abcam), rabbit anti-24p3R (1:200, Cat#ab124506, Abcam), anti-rabbit Alex 647 conjugated secondary antibody (1:200, Cat#ab150063, Abcam), goat anti-LCN2 (1:200, Cat#AF1857, R&D), anti-goat Alex 594 conjugated secondary antibody (1:200, Cat#ab150132, Abcam)

In lcn2−/− astrocytes, iNOS did not increase after hypoxia (Fig. 7c). Whereas in LCN2-overexpressing astrocytes, the elevation of iNOS expression was more significant than the control group (Fig. 7d). The immunofluorescence staining of astrocytes cultures also showed increased iNOS expression after OGD in wild-type but not in LCN2 knockout astrocytes (Fig. 8). On the other hand, the increase in iNOS expression after OGD was even higher in LCN2 overexpressing astrocytes (Figs. 9 and 10).

**Fig. 11:**
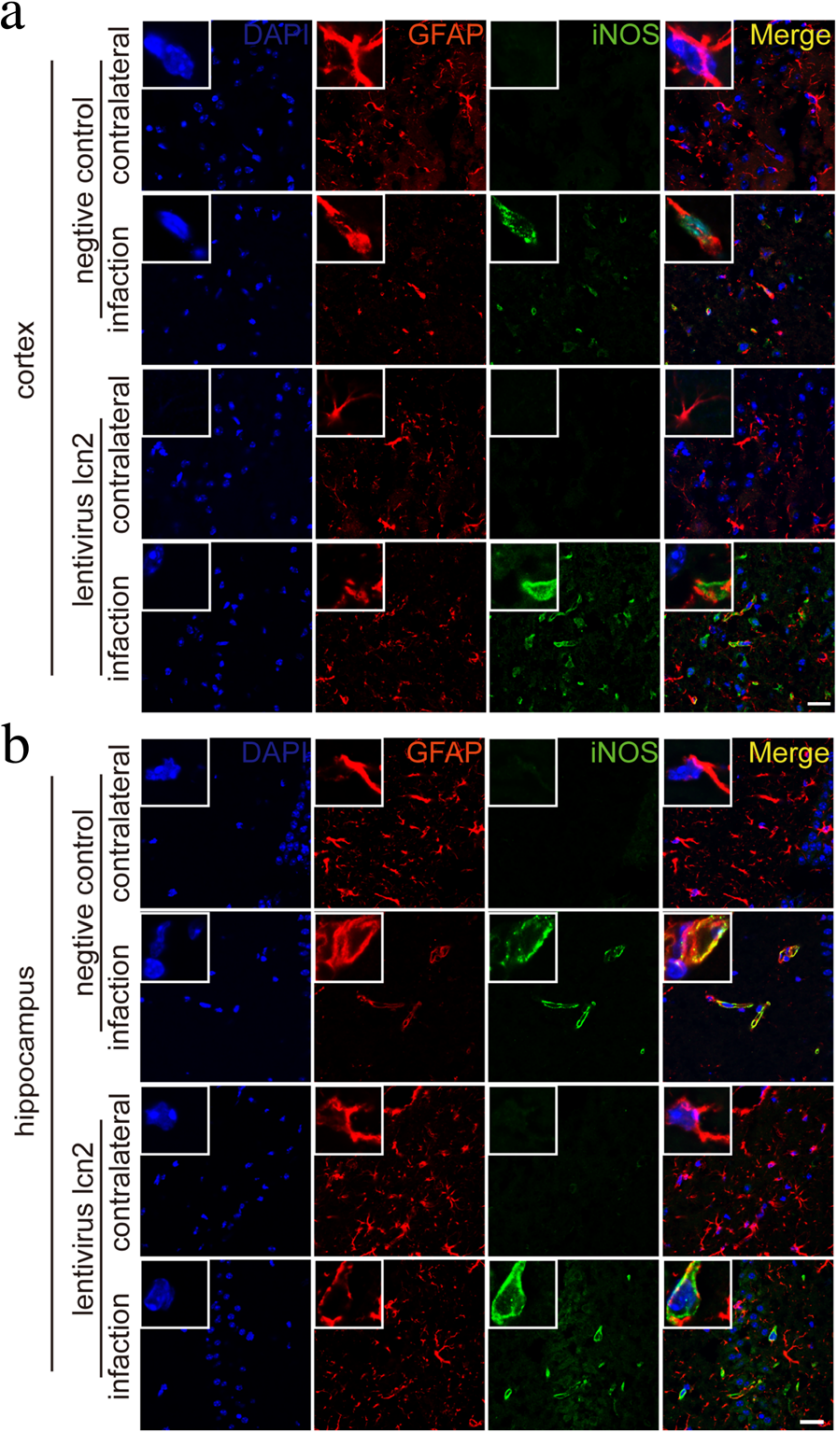
Increase of MCAO induced iNOS expression in the brain section of LCN2-lentivirus-transfected mice. The iNOS and GFAP were co-localized by immunofluorescence on brain slices of LCN2-lentivirus transfected MCAO mice. **a** LCN2 transfected mice had higher iNOS expression in infarcted cortex area than negative controls, scale bar 20 μm. **b** LCN2 transfected mice also had higher iNOS expression in the infarcted hippocampus than controls, scale bar 20 μm. Antibody: rabbit anti-iNOS (1:200, Cat#ab17945, Abcam), anti-rabbit Alex 488 conjugated secondary antibody (1:200, Cat#ab150073, Abcam), goat anti-GFAP (1:200, Cat#ab53554, Abcam), anti-goat Alex 594 conjugated secondary antibody (1:200, Cat#ab150132, Abcam)

**Fig. 12:**
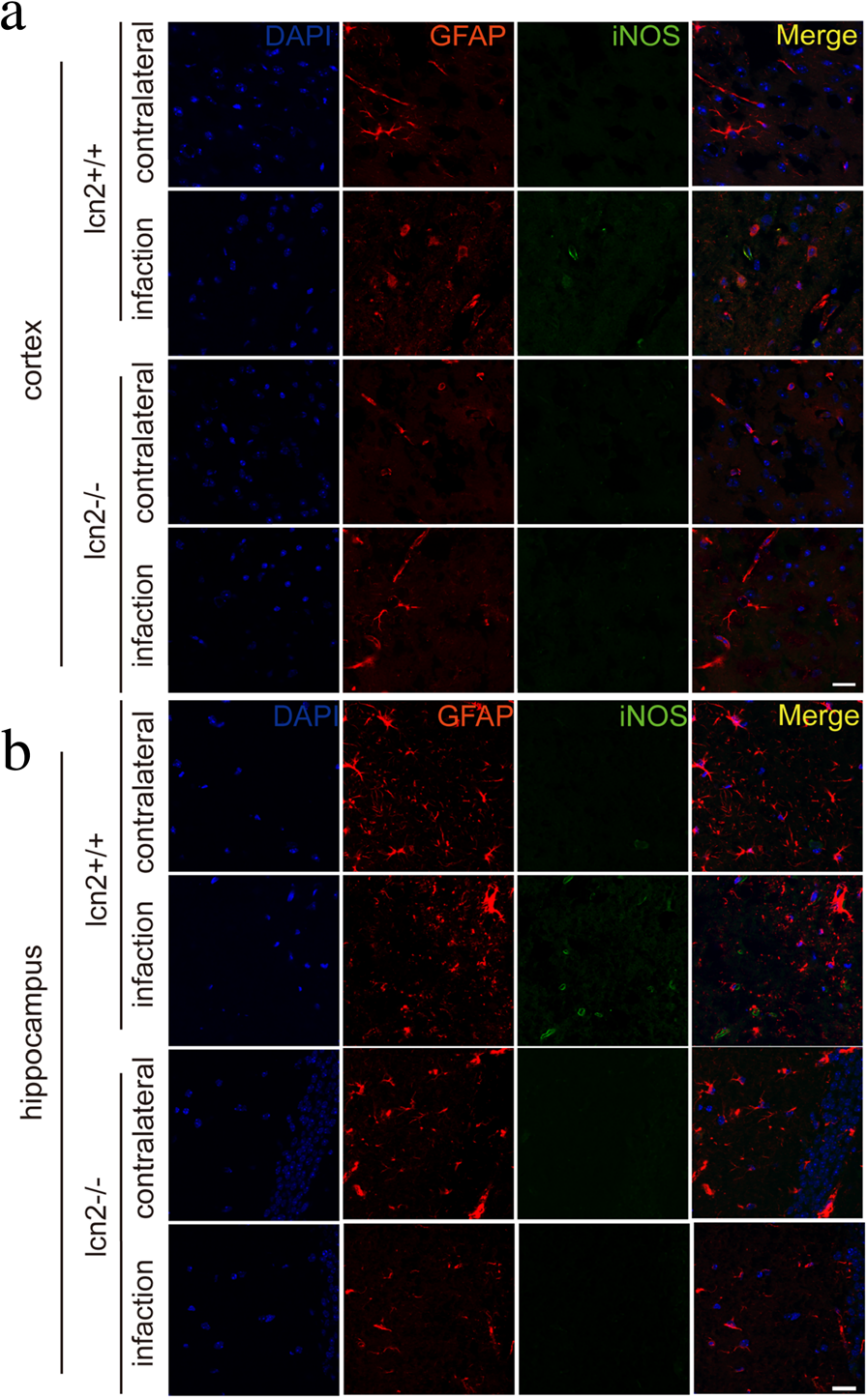
Inhibition of MCAO induced iNOS expression in brain section of LCN2-knockout mice. The iNOS and GFAP were co-localized by immunofluorescence on brain slices of LCN2-knockout MCAO mice. **a** LCN2-knockout mice did not express iNOS in the infarcted cortex, scale bar 20 μm. **b** LCN2-knockout mice had no iNOS expression in the infarcted hippocampus, scale bar 20 μm. Antibody: rabbit anti-iNOS (1:200, Cat#ab17945, Abcam), anti-rabbit Alex 488 conjugated secondary antibody (1:200, Cat#ab150073, Abcam), goat anti-GFAP (1:200, Cat#ab53554, Abcam), anti-goat Alex 594 conjugated secondary antibody (1:200, Cat#ab150132, Abcam)

In vivo, studies further confirmed the findings in middle cerebral artery occlusion (MCAO) mice models. In the brain slices, iNOS and GFAP protein were co-localized in the cells within the infarcted hemisphere and hippocampus of wild-type mice, but not in corresponding regions in the contralateral hemisphere. However, in LCN2 knockout mice models, the expression of iNOS protein on the infarct hemisphere was completely inhibited (Fig. 12). Meanwhile, LCN2 overexpression led to higher iNOS levels both in the cortex and hippocampus in the ischemic hemisphere (Fig. 11).

### Effects of LCN2 on infarct volumes and functional outcomes

**Fig. 13:**
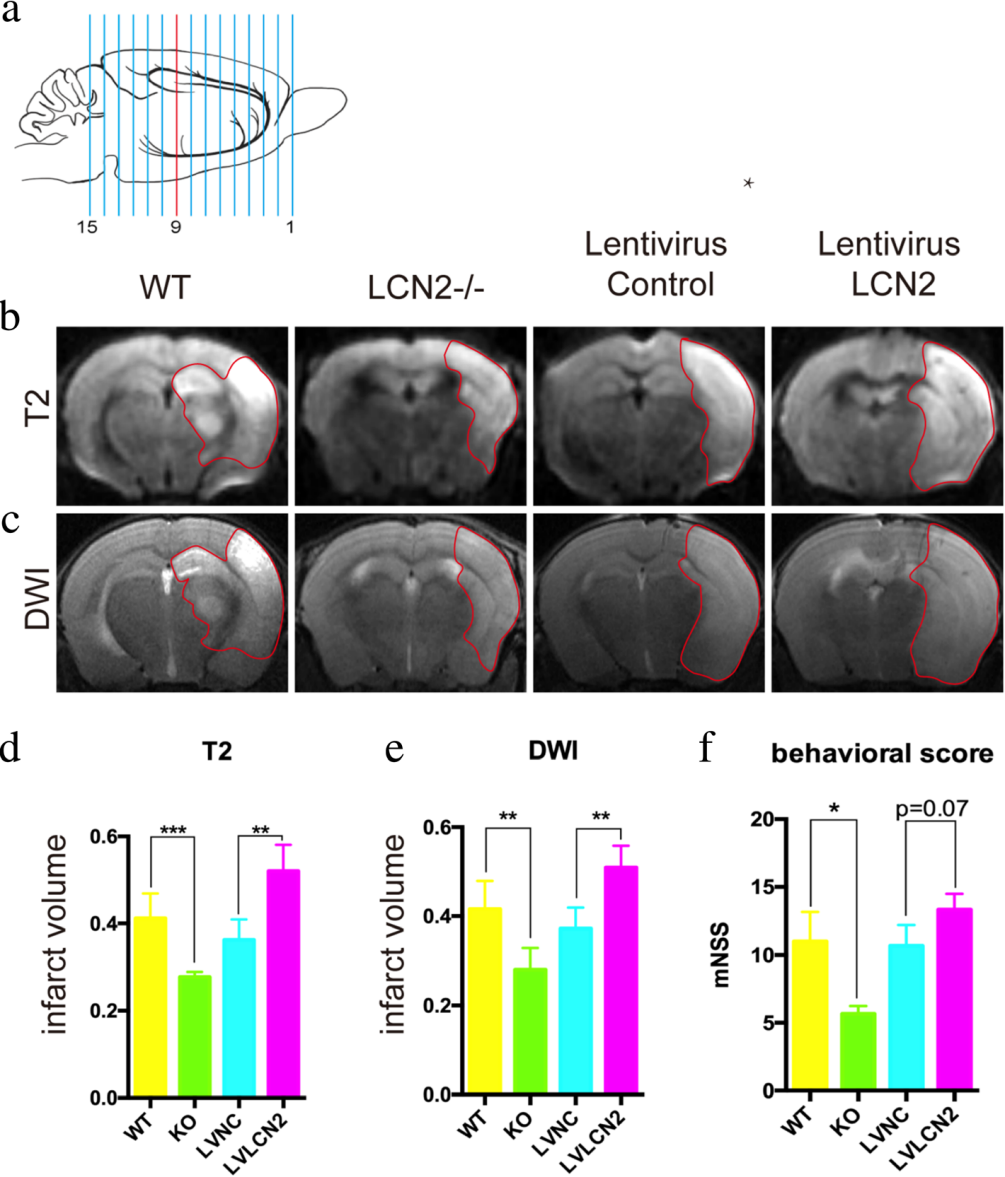
The effects of LCN2 on infarct volumes and functional outcomes after MCAO. **a** After mice being subjected to MCAO surgery, a magnetic resonance imaging (MRI) was performed. **b**, **c** On MRI T2 and DWI sequences, the LCN2-knockout mice had relative smaller infarct areas after MCAO than wild-type mice. The LCN2 overexpressed mice had larger infarct areas than negative controls. **d**, **e** The statistical results of MRI. **f** The mice were evaluated by modified Neurological Severity Score (mNSS). The LCN2 knockout mice had lower mNSS scores than WT mice (5.67 ± 0.33 and 11.00 ± 1.08, *P* < 0.01), and the LCN2 overexpressed mice tend to have higher mNSS scores than the controls (13.33 ± 0.67 and 10.67 ± 0.88, *P* = 0.07)

After MCAO, infarct volumes of LCN2-knockout mice were significantly smaller than that of wild-type mice (Fig. 12). On the other hand, LCN2-overexpressed mice had larger infarct volumes. The average infarct volumes reduced by 34.6% (from 0.423 to 0.277) in T2 sequence and by 32.6% (from 0.416 to 0.280) in diffusion-weighted image (DWI), respectively (Fig. 13a–e). Accordingly, tests with the modified Neurological Severity Score (mNSS) also showed that the neurological function improved significantly in LCN2 knockout mice, but aggravated in LCN2-overexpressed mice (Fig. 13f).

## Discussion

Functional activation has been investigated for years in microglia [3], but in astrocytes, it is a relatively new concept. The polarization of astrocytes has been widely discussed in brain trauma, which mainly focused on the recruitment of astrocytes towards the lesion, scar forming, isolating, and repairing tissue [23, 24] rather than its role in the inflammatory response after stroke. This study probed the presence and effects of functional polarization of astrocytes after cerebral ischemia and demonstrated that LCN2 played an essential role in classical activation of astrocytes and aggravated the ischemia-induced brain damage.

Effects of LCN2 on neurons have been controversial for years [25], but there was no doubt that LCN2 is a pro-inflammation factor expressed in the acute phase of ischemia [8]. Due to the complex functions and widespread receptors of LCN2, the mechanism of how LCN2 functions remained unclear [25]. The current study provided further evidence for the deleterious effect of LCN2 and also suggested effecting astrocyte polarization as a possible mechanism of LCN2 in the acute phase of cerebral ischemia.

In this study, astrocytes expressed a variety of inflammatory factors after OGD, suggesting they were involved in the inflammatory response to hypoxia. These inflammatory factors included inflammatory factors in classical and alternative activation pathways. In previous studies, many cytokines, including TNF-α, IL-1, IL-6, IL-12, IL-1β, CXCL-10, and iNOS, were used to characterize M1 type macrophages, IL-4, IL-13, IL-10, TGF-β, dectin-1, stabilin-1, ARG1, and ARG2 were used to characterize M2 type macrophages, and iNOS and ARG1 were the typical downstream molecule of classical and alternative activation pathways [26]. Among them, iNOS was described as a pro-inflammatory factor, whereas ARG1, which functioned as an anti-inflammatory factor, was one of the important products of alternative activation astrocytes [22]. Therefore, we chose iNOS and ARG1 as the representative proteins of these two pathways according to previous studies [7]. The results showed that iNOS increased greatly after ischemia, suggesting a large number of astrocytes were activated in the classical pathway. The expression of ARG1 did not change significantly after hypoxia, suggesting that the alternative activation pathway did not play a significant role at that time. The interesting thing is that mRNA levels of cytokines peaked at 6 h after hypoxia, and the iNOS protein reached the highest after 24 h. This was perhaps due to the fact that protein expression lags behind mRNA. Mild ischemic stroke was reported to induce astrocyte polarization in the lesion core [27]. In their study, the differences of b-DG, AQP4, or Kir4.1 were not observed at 12 h reperfusion, but obvious at 24 h and 48 h reperfusions [27]. It was consistent with the time for the polarization of astrocytes observed in Fig. 2, suggesting a potential involvement of LCN2 in the expression of these proteins and therefore the process of astrocyte polarization.

Selecting the astrocytes after hypoxia treatment by magnetic bead sorting offered an opportunity to investigate the two group of astrocytes separately. The iNOS(+) astrocytes and iNOS(−) astrocytes were compared on their functions to anoxic neurons. It turns out that iNOS(+) astrocytes can aggravate anoxia damage and even reduce the number of surviving neurons, while the iNOS(−) astrocytes can significantly alleviate this damage and protect the neuronal synaptic morphology relatively intact. Therefore, iNOS (+) and iNOS (−) groups of cells showed differences not only in their expression of cell markers, but also in their influences on neurons, providing evidence for the functional polarization of astrocytes.

Figure 6 showed staining in the infarcted cortex, sub-cortex, and hippocampus regions, using the corresponding regions of the contralateral hemisphere as control. The results showed that iNOS expressed in all infarction regions, especially in infarcted cortex region, but was not observed in the contralateral hemisphere. It is worth mentioning that the iNOS in infarcted cortex region was mostly co-located with GFAP. Iba-1 relatively higher expressed in infarcted hippocampus regions but was not co-located with iNOS. Though CNS resident microglia were recruited early upon stroke, the Iba-1 staining was weak in Fig. 6. It was possibly due to round Iba1-positive cells which appeared from 24 h and reached a peak at 4 to 7 days in the ischemic core [28] and (Fig. 6) was at 24 h reperfusion.

With LCN2 knockout, the expressions of cytokines in the classical activation pathway were inhibited. The expression of iNOS was almost completely inhibited in LCN2 knockout astrocytes. This indicated that the existence of LCN2 contributed to the classical activation pathway of astrocytes. Correspondingly, LCN2 overexpressed astrocytes expressed higher iNOS, which further supported the promotion function of LCN2 on the classical activation of astrocytes. According to the previous studies and the results in this study, the classical pathway of activation had more pro-inflammatory effects than the protective effect on neurons during acute ischemia, and alternative activation pathways inhibited the inflammatory reaction and mainly had a protective effect, reducing the injury of nerve cells in the acute stage [6]. In MCAO animal models, the expression of iNOS was selectively expressed in astrocytes in the infarcted area, and the expression was inhibited in LCN2 knockout animals. The interesting thing is that LCN2 was reported to express higher in the infarcted area. MRI scanning and behavioral score further indicated the protective effect on neurological function from the infarct volume and neurological function aspects. The results in LVLCN2 intracerebroventricular injection mice indicated that overexpression of LCN2 had a tendency to promote classical activation of astrocytes and increase infarct volume and neural function injury. The results of this study indicated a possibility that LCN2 may aggravate post-stroke neuronal damage by promoting pro-inflammatory activation of astrocytes. It worth mentioning that in in vivo experiment, the tMCAo model provided an environment similar to ischemic stroke, directly showed that the activation of astrocytes mainly located in the core and nearby area of infarction, along with infarcted hippocampal area, but was hardly observed in parallel contralateral regions. It indicated that astrocyte activation was directly induced by ischemia and hypoxia, instead of inflammatory mediators in cerebrospinal fluid secreted by other activated cells and provided a reason for our contralateral control. Further studies are needed to clarify the specific mechanism of LCN2 on astrocytes.

There are drawbacks in the current study. Firstly, we focused solely on the protein LCN2 and its detrimental role in brain ischemia via the regulation of astrocytes. However, the association between the immune system and the brain is a paradox and complicated [29]. Previous studies demonstrated that inflammation aggravates brain injuries in the acute phase [30, 31] but promotes tissue recovery in delayed phase [32], and recent studies further suggested that pro-inflammatory cytokines can also be beneficial during the acute phase in traumatic brain injuries [33]. In correspondence to the dual role of neuro-inflammation, astrocytes also play dual roles in brain ischemia [34]. Astrocytes exert neuroprotective effects by secreting a number of neurotrophic factors such as BDNF, contributing to brain recovery by suppressing neuro-inflammation, via the crosstalk with of TNF-alpha, IL-10 [35, 36], and IL-6 [37]. Meanwhile, the results of our study demonstrated that astrocytes not only showed inhibitory effects on post-stroke inflammation, but also promoted inflammation and aggravated tissue damage under LCN2’s regulation, which was in consistent with previous studies which suggested that astrocytes can be classified into neurotoxic A1 phenotype and neuro-protective A2 phenotype [38]. Secondly, the function of neuro-inflammation was only tested in the ischemic stroke model. However, inflammation participates in different types of injuries, including central [39–41] and peripheral nervous system [42]. Further studies are required to fully depict the role of inflammation in other neurological pathologies.

## Conclusion

This study suggested that astrocytes may be functionally polarized after cerebral ischemia. LCN2 was a necessity for the classical activation of astrocytes, which may aggravate injury in the acute phase of cerebral infarction.

## Data Availability

The datasets used and/or analyzed during the current study are available from the corresponding author on reasonable request.

## Abbreviations

ARG1: Arginase-1
CNS: Central nervous system
DMEM: Dulbecco’s modified Eagle medium
FBS: Fetal bovine serum
FJC: Fluoro-Jade C staining
HBSS: Hank’s buffered saline solution
iNOS: Inducible nitric oxide synthase
KO: Knockout
LCN2: Lipocalin-2
LV-LCN2Q: Lentivirus of overexpressed LCN2
LVNC: Lentivirus negative control
MCA: Middle cerebral artery
MCAO: Middle cerebral artery occlusion
OGD: Oxygen-glucose deprivation
PFA: Paraformaldehyde
RT-PCR: Reverse transcription polymerase chain reaction
TUNEL: Terminal deoxynucleotidyl transferase-mediated dUTP nick end labeling
